# Rethinking the Origin of the Primary Respiratory Mechanism

**DOI:** 10.7759/cureus.46527

**Published:** 2023-10-05

**Authors:** Bruno Bordoni, Allan R Escher

**Affiliations:** 1 Physical Medicine and Rehabilitation, Foundation Don Carlo Gnocchi, Milan, ITA; 2 Anesthesiology/Pain Medicine, H. Lee Moffitt Cancer Center and Research Institute, Tampa, USA

**Keywords:** spheno-occipital synchondrosis, fascia, craniosacral therapy, osteopathic manipulation, osteopathy

## Abstract

Spheno-occipital synchondrosis (SOS) is the joint regarded as the most important foundation for understanding cranial osteopathy and craniosacral therapy. SOS is the origin of the primary respiratory mechanism (PRM), a movement between the posterior surface of the body of the sphenoid bone and the anterior surface of the base of the occipital bone. From the PRM perspective, an alteration of the position between the two bone surfaces would create cranial and/or craniosacral dysfunction. These positional alterations of the SOS (in adults and children) would determine specific and schematical movements of the bones of the entire skull, whose movements are recognizable by palpation by trained operators. PRM expression is influenced by other elements, such as movement of the cranial bones, inherent movement of the central nervous system, cyclic movement of cerebrospinal fluid (CSF), mechanical tension of the cranial meninges, and passive movement of the sacral bone between the iliac bones. The article reviews the most up-to-date information on the evolution of cranial sutures/joints and meninges in adulthood, the fluctuations of the CSF, brain, and spinal mass movements. Research should reconsider the motivations that induce the operator to discriminate the palpable cranial rhythmic impulse, and probably, to rethink new cranial dysfunctional patterns.

## Introduction and background

Cranial osteopathy and craniosacral therapy were born from the observation and intuition of an osteopathic student at the American School of Osteopathy, back in 1898; the student's name was William Garner Sutherland [1]. Sutherland acquired the title of doctor of osteopathic medicine (DO)** **and devised the concept of the primary respiratory mechanism (PRM), from which the theory of movement of the bones of the skull is based, and for which the presence of cranial movements is included in codified patterns. In Sutherland's book published in 1939, the PRM hypothesis is illustrated, which is based on and conditioned by: the inherent movement of the central nervous system and spinal cord; the fluctuation of the cerebrospinal fluid (CSF); the mechanical tension arising from the cranial meninges; the movement of the sacral bone between the iliac bones; movements of the bones of the skull thanks to the sutures [2]. These movements are not voluntary but would depend on the fluctuations of the cranial fluids, and on the cranial and spinal dural elastic response to the passage of these fluidic oscillations [3]. These cranial rhythms would be slower than the thoracic respiratory and heart rate rhythms: 15.5 cycles in one minute of chest motion with plethysmograph, and 12.8 cycles in one minute of skull motion measured using an oscillograph [3]. These cranial oscillations would be determined by pressure variations of the central and peripheral nervous system, imposed by changes in the amount of fluids, in a cyclical and rhythmic manner that can be recorded by machinery.

The fact that there are pressure changes within the skull, and to prevent such fluctuations from harming brain function, the skull should create space for better distribution of fluids. According to the PRM theory, there would be changes in the anteroposterior and lateral diameters of the skull, palpable by trained operators, thanks to the mobility of the cranial sutures [2,4]. If the pressures increase the skull dilates, if the pressures decrease the skull narrows; this protection mechanism, created by the "Master Mechanic" (as Sutherland wrote, that is, Nature), is identified as a flexion and extension of the skull, respectively [4,5]. The movement is in millimeters (0.0127-0.0254 millimeters on average), always measured with an oscillometer with the subject supine and calm [3]. 

The article reviews the most current scientific data on the ossification of spheno-occipital synchondrosis (SOS), passing through the embryological origin of the sphenoid and occiput, as well as the time of obliteration of cranial sutures over the course of age. The text highlights the need to observe the phenomenon of cranial bone movement under a different intellectual lens, compared to 1940, when the study and applications of osteopathic cranial practice began in a university setting [6]. Additionally, the article reviews the components that would influence PRM, filtering them with current scientific information. The aim of the article is to stimulate osteopathic and craniosacral research to reconsider the motivations that lead the operator to discriminate the palpable cranial rhythmic impulse (CRI) in the adult, and probably, to rethink new cranial dysfunctional patterns, with respect to physiological and non-physiological patterns of physiological systems devised by Sutherland.

## Review

Flexion-extension of the skull

**Figure 1:**
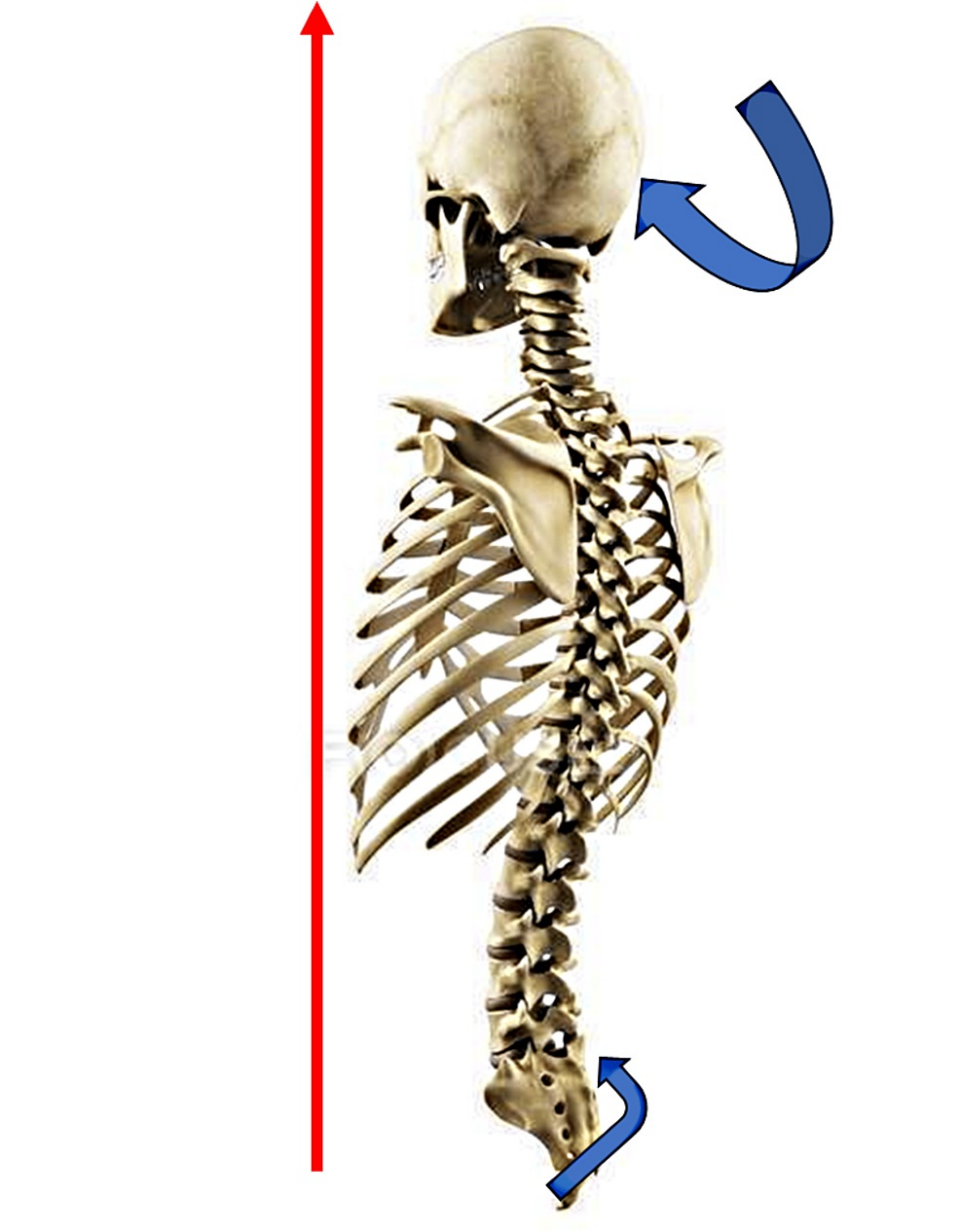
Cranial and sacral movements during craniosacral flexion The red arrow indicates the cranial direction of the craniosacral system during flexion of the sphenobasilar synchondrosis. The cranial blue arrow indicates the tilting movement of the occipital bone (as if it were re-entering), while the blue arrow near the sacral bone indicates the traction movement of the base of the sacral bone upwards or nutation. The exact opposite movement occurs during craniosacral extension. The figure is owned by Bruno Bordoni.

According to the osteopathic and craniosacral perspective, the flexion-extension of the skull would mainly take place, thanks to the movement of the spheno-occipital synchondrosis (SOS) joint. The latter is seen as two cogwheels, whose movements allow to obtain flexion (SOS in elevation and dorsal face with greater convexity), and extension (SOS in depression and ventral face with greater convexity) [5]. Flexion affects the ethmoid bone and the vomer (these two undergo backward, downward, and outward advancement), with an outward and forward thrust of the greater wings of the sphenoid, and a lateralization of the angles of the frontal bone [2]. The palatine bones undergo an outward and backward movement, the zygomatic, maxillary, and mandibular bones and the temporal bones would undergo an external rotation [4]. The parietal bones tend to move externally, with the interparietalsuture having a caudal vector; basically, the uneven bones tend to undergo a flexion-extension movement on the sagittal plane, while the even bones tend to undergo an external-internal rotation [4]. If the passive movement of the cranial bones is present with specular entities (velocity, amplitude, and rhythm), the person would not show pathological signs (Figure 1) [2].

Embryology of the occipital bone and the sphenoid bone

The clinician should know the embryological origin of the skull bones and the bone evolution during growth, in order to obtain a broader view of the PRM phenomenon [7]. The occipital bone is among the first bones of the skull to develop, with mesodermal and ectodermal origins [8]. The basioccipital or basilar part is the union of the first four cranial vertebrae, with cartilaginous derivation (chondrocranium); the newborn presents the basilar area in conjunction with the lateral occipital areas through synchondrosis (right and left), which joint ossifies completely by the fourth year of age [8]. The basilar part will have articular relationships (synchondrosis) with the basisphenoid becoming the clivus [8]. The supraoccipital area or squamous part originally consists of three portions: central, right, and left. The central segment is delimited by vertical sutures, which range from the interparietal-supraoccipital suture to the posterior border of the foramen magnum [8]. The squamous part in its inferior and lateral portion constitutes the posterior cranial fossa; superiorly and laterally it joins the parietal bones through the lambdoid suture and the occipito-mastoid suture with the temporal bone [8].

The interparietal suture has a membranous origin (desmocranium), while the supraoccipital suture has a cartilaginous derivation [9]. The exoccipital part or condylar area forms the lateral margin of the foramen magnum; this segment at birth has synchondroses that unite it to the basioccipital and the occipital squama, which synchondroses ossify rapidly [8]. The condylar area has a cartilaginous origin [9]. The mendosal suture parallel to the lambdoid suture can be found; the first begins to ossify in the fetus, until it disappears completely between the second and fourth years of life or persists in adults [8]. The development of the cartilaginous areas takes place after the formation of the vascular and nerve pathways [10]. The sphenoid bone formation has a large cartilage-like component such as the ala temporalis, alar process, presphenoid, basisphenoid, and orbitosphenoid, while the ascending lamina and the greater wing have a membranous origin [9, 11,12].

The presphenoid and basisphenoid will form the body of the sphenoid; the lesser wings and the anterior area of the sphenoid body will derive from the orbitosphenoid, while the dorsum sellae and sella turcica will derive from the basisphenoid [12,13]. The medial pterygoid process derives from a membranous and cartilaginous formation, while the lateral portion of the medial pterygoid process has an exclusively membranous derivation [13]. The lateral pterygoid process has a purely cartilaginous origin. From the ala temporalis and the alar process will derive the foramen ovale, the foramen rotundum and the greater wings [13]. The basisphenoid and orbitosphenoid arise from the mesoderm, while the presphenoid and alar processes arise from the ectoderm; the sphenoid is formed between the fourth and eighth weeks of gestation [14,15]. The sphenoid connects the neurocranium with the viscerocranium.

Maturation and ossification of the spheno-occipital synchondrosis

The occipital bone is part of the skull vault or calvaria (together with the frontal, parietal and temporal bones), while the sphenoid and occiput form the skull base (together with the ethmoid, frontal and temporal bones) [9]. In particular, the sphenoid bone and the occiput form the posterior cranial fossa [9]. SOS plays an important role in the longitudinal growth of the skull; an alteration of the physiological development can negatively influence the shape of the skull, inducing hypoplasia of one side of the face and microcephaly [16]. Synchondrosis develops in a different way than in a long bone. Bipolar growth plates, which create areas of growth of chondrocytes in opposite directions, and ossification formation by osteoblasts are seen [16]. Complete ossification or synostosis of SOS occurs between the ages of 16-18 years in the human model[16]. Until adulthood is reached, SOS remains patent. A retrospective study of 638 cadavers (10-25 years of age), highlighted an average age of complete ossification of the SOS around 17 years; moreover, females begin the ossification process about two years earlier than males [17]. Ossification develops from the superior area of the SOS, eventually involving the inferior portion [17]. Another forensic study using living MRIs evaluated 208 participants (ages 5-30 years). The study lowers the age of detection of complete ossification, where in the male it is 15 years, while in the female the SOS ends at the age of 13 [18]. A forensic investigation using computed tomography (CT) scans with 500 cadavers and an age range of 5-25 years, revealed that complete ossification of SOS occurs by 18 years of age [19]. Another study with 198 cadavers (8-26 years) described complete ossification of SOS for men between 17 and 21 years of age, whereas for women it occurs before approximately 1 to 2 years of age [20]. Another forensic study employed 147 cadavers (12-30 years), where regardless of sex, complete fusion of the SOS is found around 20 years of age, with ossification onset about 3 years earlier in females [21].

Forensic and anatomical studies

**Figure 2:**
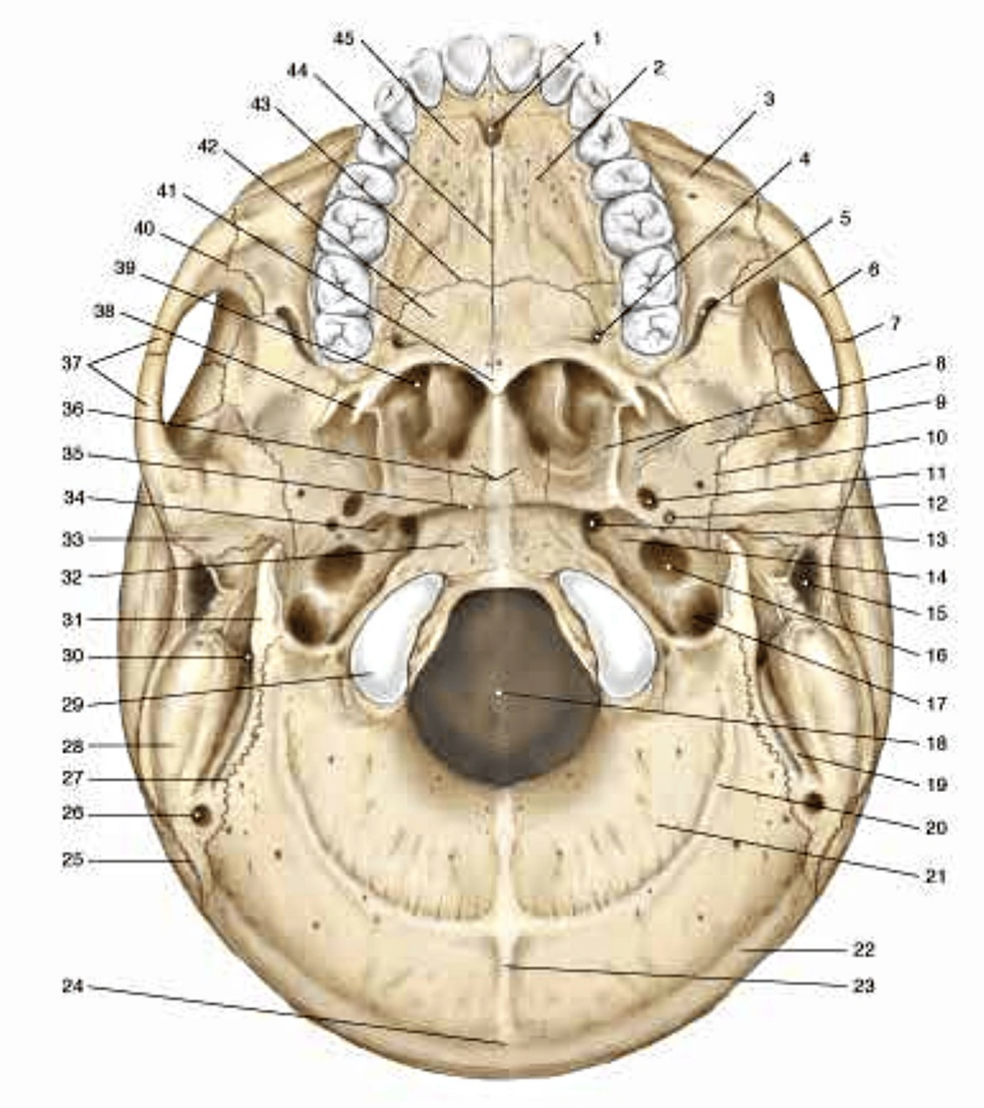
Anatomical image highlighting the lower external area of the adult skull. 1 Incisor forum; 2 Maxillary bone; 3 Zygomatic process of the maxilla; 4 Greater forum palatine; 5 Lower orbital fissure; 6 Zygomatic bone; 7 Temporozygomatic suture; 8 Pterygoid process; 9 Great wing of the sphenoid; 10 Suture sphenosquamosal; 11 Oval forum; 12 Spinous forum; 13 Lacerated forum; 14 Pyramid of the temporal; 15 External acoustic meatus; 16 Carotid canal; 17 Jugular forum; 18 Foramen magnum; 19 Mastoid notch; 20 Inferior nuchal line; 21 Nuchal plane; 22 Superior nuchal line; 23 External occipital crest; 24 Inion; 25 Lambdoid suture; 26 Mastoid forum; 27 Occipitomastoid suture; 28 Mastoid process; 29 Occipital condyle; 30 Stylus mastoid forum; 31 Styloid process of the temporal; 32 Basal portion of the occiput; 33 Mandibular fossa; 34 Groove of auditory tube; 35 Spheno-occipital synchondrosis; 36 Wings of the vomer; 37 Zygomatic arch; 38 Pterygoid hook; 39 Inferior nasal concha; 40 Zygomatic maxillary suture; 41 Inferior nasal spine; 42 Horizontal lamina of the palatine; 43 Transverse palatine suture; 44 Median palatine suture; 45 Palatine process of the maxilla. Reproduced with permission from Anastasi et al. [23].

Considering forensic and anatomical studies starting from 1960, the slight differences in finding the complete closure between the two bones depends on the geographical area where the research was carried out; rare exceptions remain, where SOS is still patent [22]. The ossification or closure of the SOS is a full bony fusion between the occiput and the sphenoid bone [22]. The closure of the SOS, through instrumental investigations, could be invisible or be identified as a "fusion scar"; the latter is a radiodense line reflecting a superficial groove where the SOS was [22]. When SOS is ossified it becomes a single bone, like the diaphysis of a tibia: there is no flexion-extension or any other movement (Figure 2) [23].

Wormian bones

It should be remembered, however, that there are many adults (8%-80% of the population) who present with Wormian bones, i.e., more bones than the number of cranial bones recognized as physiological (28 flat and irregular bones) [24-26]. These bones form and mature within sutures and can be considered as anatomical or syndromic variants, as in patients with osteogenesis imperfecta or bone dysplasia, and pathologies related to genetic disorders [24]. The presence of additional bones creates different sutures and in different locations than in a skull without these bones. The precise mechanism of formation of these bones is not known, moreover, the presence of Wormian bones found outside the skull does not always coincide with intracranial bone formation [26]. We do not have precise data on the life span of ossification of these bone anomalies.

Ossification of the cranial sutures

One mechanism that would help explain PRM is the movement of the bones of the skull. To ascertain whether this event occurs, we need to review the information related to the ossification-closure of the cranial sutures. From an anatomical point of view, if the cranial sutures are ossified, movement between the bones is absent [27]. Assessment of suture ossification is done by evaluating the amount of closure; grade zero equals a patent suture, grade one indicates that the suture is more than 50% open, grade three means that more than 50% of the sutural connection surface area is closed, and grade four is complete ossification [27]. A study evaluating 285 cranial vaults in adult cadavers found that the sagittal suture ossified in 85.72%, the coronal suture closed in 62.64% of skulls, and the lambdoid suture obliterated in 48%, 35% [27]. Ossification occurs gradually throughout life, and the extracranial sutures (closed or patent) do not always reflect the evolution of the intracranial sutures, or the closure does not involve the entire thickness of the bone, making it difficult to draw conclusions on such non-homogeneous adaptation [28,29]. Probably, it is the variability of the age of the skulls examined; the greater the obliteration, the greater the age of the person [28,29].

Generally, the complete closure of the sagittal suture is around the age of about 51, starting ossification from about the age of 13 [28]. A retrospective study on 390 patients and the use of computed tomography (CT) evaluated the evolutionary behavior of the sutures from the age of 1 month up to the age of 90 years [30]. The coronal suture begins to ossify from about age 14 and continues to obliterate beyond 80 years of age [30]. The lambdoid suture, according to a study with CT and 230 patients, begins to ossify around the age of about 27, to continue beyond the age of about 70 [31]. Other studies identified the onset of obliteration at about 4-15 years of age, with complete ossification at about 42 years [27,30,32]. Further data are needed for a more comprehensive determination of the evolution of the lambdoid suture. The metopic suture of the frontal bone ossifies completely by 7 years of age; in some rare exceptions, part or all the suture is not closed and takes the name of "sutura frontalis (metopica) persistens” [33].

The frontosphenoidal suture begins to ossify from the beginning of about 30 years, to completely obliterate towards about 60 or 80 years [30,32]. The frontonasal suture ossifies completely by approximately 60 years of age [34]. The frontomasillary sutures begin to obliterate only after about 70 years of age, while the frontozygomatic suture begins to close after about 80 years of age [34]. Continuing with facial viscerocranium sutures, the sphenozygomatic suture and the frontoethmoidal suture close by age 60; the zygomomaxillary suture is not completely ossified in people over 70 years old, as is the nasomaxillary [34]. The pterygomaxillary suture between the sphenoid bone and maxillary process remains patent until over eighty years of age, although the data we have are very scarce [35]. Sphenethmoidal synchondrosis is obliterated between approximately 15 years and 30 years of age [30,34].

The sphenoparietal is completely closed by the end of adolescence; the sphenotemporal should be closed by the end of approximately 60 years of age [30,34]. The occipitomastoid suture is obliterated by 16 years of age; the sphenopetrosal suture is ossified by age 30 [34]. The zygomatic-temporal suture begins to obliterate in the seventh decade of life; the suture between the bones of the nose closes by age 30 [33]. For nasoethmoid sutures, septovomerine and lacrimal bone ratios have no data. The intermaxillary suture is closed by 20 years of age, and ossification of the interpalatine and transverse palatal sutures starts from adolescence, but they appear to be completely closed only around about 60 years of age [32,36-38]. We have no data on the maturation of the palatoethmoidal sutures. The petrosquamous suture in adulthood is obliterated [38]. Parietomastoid sutures obliterate within the sixth decade of age [33]. The different bones that ossify become a bony continuity and cannot move.

The movement of the brain mass and spinal cord

According to the PRM model, the intrinsic movement of the brain mass and spinal cord would cause cyclical fluctuations of the CSF, with specific movement patterns of the SOS, cranial bones, and sacrum [39]. We must remember that theory is an idea to try to explain a phenomenon that is not fully understood or proven [40]. There are several reasons that lead the nervous system to make fluctuations. The extracellular matrix (EM) surrounding each cell has piezoelectric properties [41]. The molecules that make up the EM undergo many morphological deformations, which changes in shape determine an alteration of the electric polarity, generating an electric potential. The altered electric potential generates the movement of electrons, which movement leads to the creation of an electric field. The electric field encounters other cellular electric fields of each tissue, creating an electromagnetic interaction and electromagnetic waves, which propagate throughout the body [42].

Electromagnetic waves can influence the velocity of EM fluids, a mechanism defined as electro-osmosis of non-Newtonian fluids [43,44]. Could EM fluid velocities produce possible palpable rhythms by experienced operators? We do not know. Electromagnetic waves also derive from all neural cells, which generate periodic stereotyped spikes or synchronous rhythmic oscillations [45,46]. These waves are classified according to amplitude and rhythm; beta band (13-30 Hz), theta (4-12 Hz), gamma (30-90 Hz), alpha (8-15 Hz) and delta band (<4 Hz) [47]. Each frequency can activate neural areas for specific tasks [47]. The nervous system is involved in different rhythms and oscillations. Could one or more of these rhythms be related to the cranial rhythmic impulse (CRI)? We do not know. As Viola Frymann herself states, the possibility of palpatory illusion exists; this means that the operator who is palpating the skull does not always actually feel the cranial movement [3]. Probably, it is the vibrations coming from the operator's hands that produce a "palpatory deception".

Breathing and heartbeat

The central nervous system and spinal cord undergo caudal and cranial motion in a cyclic fashion. During an inspiration, the brain mass tends to undergo a non-homogeneous cranial movement of about 2-3 millimeters, with increased anteroposterior and lateral diameters, and a slight torsion, while with expiration the opposite phenomenon of caudalization occurs [48]. Likewise, the movement of the spinal cord mirrors the movement of the brain mass. During a systole, intracranial pressures increase with vasodilatation of the arterial vessels; to compensate for this increase in blood volume, the brain mass undergoes a caudal movement and a slight twist, thus reducing the intracranial pressure [49]. The brain mass always undergoes this movement in a non-homogeneous way.

As for the respiratory cycle, depression occurs mainly for the diencephalon, brain stem, and cerebellar tonsils (0.1-0.5 millimeters), particularly, in the very early systolic phase, and with different rates (250-500 milliseconds) depending on the brain area [49,50]. The reverse phenomenon occurs with the diastolic cycle. For more details, we recommend reading the review by Almudayni [49]. The tension suffered by the cerebral mass due to these respiratory and cardiac movements is different; the cortex area is affected by the greatest strain, while the periventricular white matter area is the area that undergoes the least strain [51]. The movement of the brain mass and spinal cord is greatest with the respiratory cycle, while it is fastest with the cardiac cycle; more CSF moves with the breath, but more slowly, while with systole, CSF moves faster but less [49,50,52]. Overall, we can intuit that the movement of the central and peripheral nervous system is chaotic (breathing associated with heartbeat). We don't know if these are the rhythms felt by trained operators.

Movement of the cerebrospinal fluid

The inflow and outflow of the CSF do not follow a homogeneous cycle, and it is not possible to create a precise pattern of the rhythm and amplitude of the movement it undergoes. CSF can be found in the ventricles, in the subarachnoid space, where it will contact the perivascular space (in contact with the extracellular fluids), in the interstitial space (in communication with the ventricular wall), and within the cellular fluids [53]. The quantity produced in 24 hours is about 500 milliliters, with a synthesis of about three to four times a day, with circadian and age-based variables [53]. One of the foundations of craniosacral therapy is the claim that CSF is produced cyclically by the choroid plexus and is one of the reasons for pulsatility [52]. Current research casts strong doubts on whether the choroid plexus is the source of CSF synthesis; one of the hypotheses is the production by the capillary-astrocyte complex, filtering the interstitial fluids. Furthermore, it is the cardiac rhythm and the movement of the diaphragm muscle that prompts the inflow-outflow of this fluid [53]. CSF always moves in a bi-directional manner, with forward and backward movements; this fluid enters and leaves the nervous system in a chaotic manner, without a precise pattern [53,54]. It cannot be the CSF that conditions the CRI felt by osteopaths and manual therapists.

CSF has different exit routes from the skull. CSF follows the olfactory nerve pathways anteriorly (via meningeal lymphatic vessels) towards the ethmoid bone, penetrating the cribriform plate [55,56]. It follows the lymphatic pathways of the maxillary and mandibular areas, up to the lymphatic pathways of the neck; and follows the extracranial exit pathways of the cranial nerves and spinal pathways (subarachnoid space) and venous pathways. Finally, it is absorbed by various tissues, by the blood, and partly returned to the central nervous system or excreted by the kidneys [57]. The movement of CSF in the spinal cord subarachnoid space is turbulent and nonlinear [58]. This behavior not only derives from the different pressure stresses deriving from the cardio-respiratory cycle, but also from the different dural compliance of the spinal cord and the subarachnoid spaces [59,60]. The non-homogeneity of the movement of the CSF at the central and peripheral level, in theory, should not offer a pattern perceptible by palpation.

Cranial meninges

The cranial meninges play an important role in the maturation of the sutures, thanks to the mechanical tension of the intrinsic structure of which they are constituted (collagen, elastin, fibroblasts, epithelial cells, stem and immune cells, and a mucopolysaccharide-water matrix), and the genesis of many biochemical paracrine factors towards the sutures themselves, up to ossification [33]. The dura mater (pachymeninx) has isotropic and anisotropic viscoelastic characteristics; and protects neural functions from possible trauma [61,62]. A study with cadaver skulls (from 2 to 94 years), analyzing the dural elastic properties, showed that the viscoelastic capacity does not change according to the anatomical position or from one side to another; it does not change with respect to gender [62]. What varies is the dural thickness and the viscoelastic capacity with advancing age, where it increases and decreases, respectively [62]. Such variables are also found in the spinal cord [63].

The arachnoid and pial layer (leptomeninges) or pia-arachnoid complex (PAC) play a role as mechanical shock absorbers for the protection of the central and peripheral nervous systems [64]. Unlike the dural layer, PAC has non-heterogeneous viscoelastic properties; there is greater stiffness where blood vessels are present within, and there is greater thinning near the cerebellum [64]. The meninges that are involved in understanding the CRI are falx cerebri, tentorium cerebelli and falx cerebelli, sella turcica or diaphragma sellae. The falx cerebri tends to ossify physiologically in the superior convex portion or in the first anterior half (more rarely in the posterior portion), during the adult stage (from 25 years onwards), for a maximum of 0.7-7% of the population; the finding of this change always occurs casually, while it is more frequent to find this structural ossification in symptomatic patients with vascular or metabolic pathologies [65-70]. There does not seem to be a variability of ossification with respect to gender [68].

**Figure 3:**
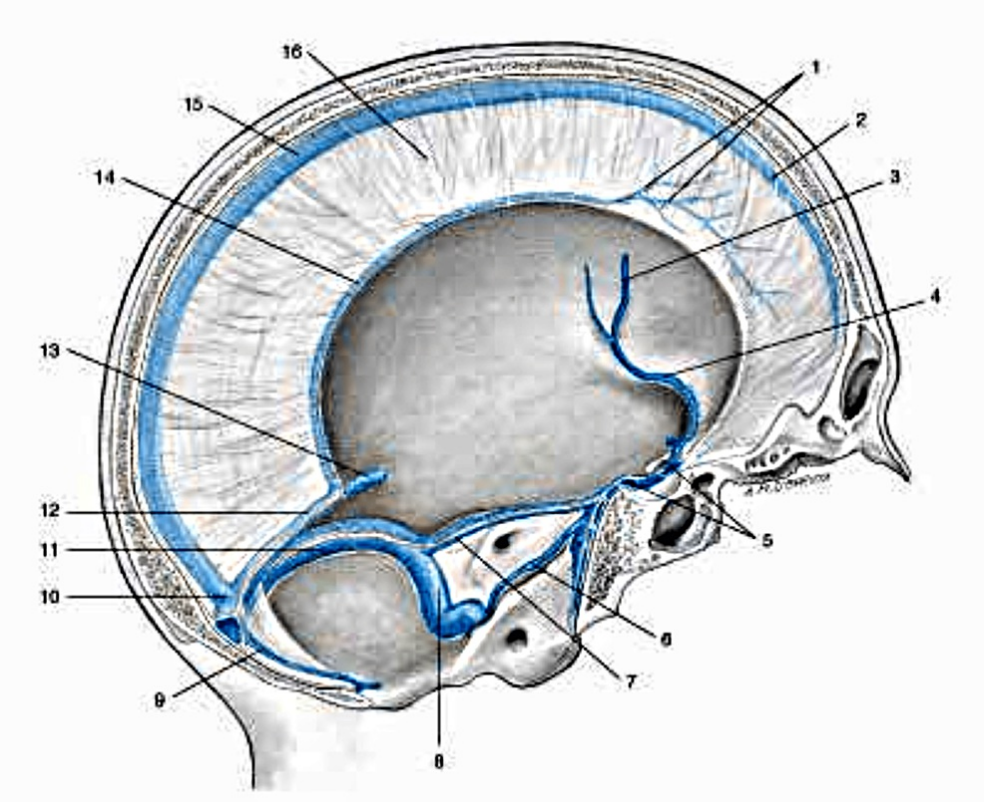
The most important venous pathways of the skull and the cranial meninges. 1 Veins of the dura mater; 2 Superior sagittal sinus; 3 Left middle cerebral vein; 4 Left sinus sphenoparietal; 5 Intercavernous sinus; 6 Left inferior petrosal sinus; 7 Left superior petrosal sinus; 8 Left sigmoid sinus; 9 Occipital sinus; 10 Confluence of sinuses; 11 Left transverse sinus; 12 Straight sinus; 13 Great cerebral vein (of Galen); 14 Inferior sagittal sinus; 15 Superior sagittal sinus; 16 Falx cerebri. Reproduced with permission from Anastasi et al. [23].

We have less data or no information on the tentorium cerebelli (case reports only) and on the other meninges. The tentorium can be partially absent (agenesis) or partially formed (hypoplasia), without necessarily the presence of neurological pathology [71-74]. The tentorium can be present in greater numbers (duplication or triplication), without necessarily syndromic signs, as well as the falx cerebelli [75-77]. The sella turcica can ossify, particularly in the dorsal portion, or only some dorsolateral portions [78,79]. With advancing age, the cranial meningeal structures can and could change their histology and function, becoming less elastic. We need more data to fully understand how the cranial meninges fit into the adult CRI concept (Figure 3) [23].

Spinal meninges and sacrum movement

The dural layer of the spinal cord meninges can undergo ossification with age. Probably, this adaptation could be a secondary phenomenon related to the ossification of the soft tissues of the spine, such as the ligamentum flavum [80,81]. Ossification of the latter is usually syndromic, like myelopathy [82]. Similarly, ossification of the posterior longitudinal ligament, and anterior longitudinal ligament leads to ossification of the spinal dural layer and symptoms [83]. In the adult, considering that no tissue maintains the same functional characteristics compared to the pediatric age, the dural meninges of the spinal cord could become less elastic. The occipital bone connects to the sacrum indirectly via the dural neural tube [7]. The movement of the sacral bone between the iliac bones (sacroiliac joint, SIJ) would have depended in a one-to-one manner, according to the perspective of craniosacral therapy on the movement of the SOS. Ossification of the posterior longitudinal ligament can cause ossification of the sacroiliac joint secondarily in adulthood [84]. SIJ can undergo a non-syndromic physiological synostosis with aging [85,86]. The sacrum moves between the iliac bones (maximum two millimeters), and if this movement is limited or exaggerated, symptoms such as local or referred pain arise [87]. We can strongly hypothesize that SOS in adulthood is unable to move the sacrum, contrary to the concept of PRM.

Future directions

The human body is a generator of multiple rhythms, from the constant contraction and relaxation of the single cell to the heartbeat, from the passage of body fluids to the breath. Rhythms are the different languages that cells and tissues have at their disposal to adapt [88,89]. These constant rhythms create involuntary swaying of the body, which affects walking or involuntary movement of the head when supine [90,91]. Which of these rhythms is felt by the operator who palpates the bones of the skull? According to some authors, the therapist and osteopath, when palpating the skull in search of the CRI, feel a low-frequency cyclic oscillation that reflects the blood flow velocity, or the Traube-Hering-Mayer waves (THMw), which would reflect the activity of the sympathetic system [92,93]. The problem arises from the fact that these waves have been inferred and measured on an animal model, with an open chest and with a paralyzed diaphragm [3]. Is it possible to compare the THMw to understand the CRI phenomenon? Probably not.

Some authors report a good inter-rater reliability between operators and cranial palpatory perception [93-95]. Other authors have failed this inter-rater reliability [96,97]. A recent systematic review reaffirmed that there is no consistent evidence on the existence of CRI and the efficacy of craniosacral therapy [98]. Some authors report cranial movements measurable by magnetic resonance imaging, with changes in cranial bone volume with a mean of 0.898 mm [99]. However, we know that the same magnetic resonance can induce, through stimulation of the vestibular area, involuntary millimeter movements of the head [100]. In light of the information presented in the article, all movement patterns perceived by palpation, which movements would be related to SOS (flexion, extension, compression, torsion, sidebending/rotation, vertical or lateral directions), must necessarily change. Furthermore, the factors involved in the CRI, which would explain this phenomenon, should be seriously re-evaluated, eliminating the school imprinting of the last century. Probably, we should rethink the new cranial patterns palpated by the operator, setting aside the previous patterns.

Craniosacral therapy and related research should also take into consideration other factors hardly used to obtain new explanations, such as the glymphatic system, the hysteretic mechanical behavior of the nervous system, and the vascular system of the bones. Sutherland defined the skull as "an intricate mechanism", while we can read in an article by Upledger the phrase "Absence of proof does not necessarily indicate proof of absence" [4,101]. However, we must be objective with the literature that we have available and strongly advise abandoning the reasons that would explain the movement of the skull, as well as the palpation patterns of the last century in the adult subject.

## Conclusions

The craniosacral therapy devised by the American osteopath Sutherland bases its theoretical foundations on the movement of the spheno-occipital synchondrosis (SOS) joint and other phenomena, such as the intrinsic oscillations of the nervous tissue, the movement of the cerebrospinal fluid (CSF), the mechanical tension resulting from the cranial meninges, the movement of the sacral bone between the iliac bones, and the movements of the skull bones thanks to the sutures. The article reviewed the most recent information on the maturation of the sutures of the SOS and cranial bones, the behavior of the CSF, the maturation of the cranial meninges, and the evolution of the sacroiliac joint. We can strongly advise abandoning the absolute certainty of the validity of the mechanisms devised by Sutherland and looking for new motivations and new methods of palpation, with respect to what is palpated by expert operators, freeing oneself from the school imprinting of 1940.
